# *CYP24A1* DNA Methylation in Colorectal Cancer as Potential Prognostic and Predictive Markers

**DOI:** 10.3390/biom15010104

**Published:** 2025-01-10

**Authors:** Ru-Hua Zhou, Lei Li, Qing-Jian Ou, Yi-Fan Wang, Yu-Jing Fang, Cai-Xia Zhang

**Affiliations:** 1 Department of Epidemiology, School of Public Health, Sun Yat-sen University, Guangzhou 510080, China; zhourh6@mail2.sysu.edu.cn (R.-H.Z.); lilei65@mail2.sysu.edu.cn (L.L.); wangyf369@mail2.sysu.edu.cn (Y.-F.W.); 2 State Key Laboratory of Oncology in South China, Guangdong Provincial Clinical Research Center for Cancer, Sun Yat-sen University Cancer Center, Guangzhou 510060, China; ouqj@sysucc.org.cn

**Keywords:** *CYP24A1*, DNA methylation, prognosis, colorectal cancer, immune infiltration, leukocytes

## Abstract

The DNA methylation of *CYP24A1* can regulate its gene expression and may play a role in the occurrence and progression of colorectal cancer (CRC). However, the association between *CYP24A1* DNA methylation and the prognosis of CRC patients has not yet been reported. In this study, differential methylation analysis was conducted in both blood and tissue cohorts, and differential expression analysis was performed in the tissue cohort with in vitro validation. GO and KEGG enrichment analyses were performed on *CYP24A1*-related genes. A correlation between *CYP24A1* promoter methylation and its gene expression was explored. Kaplan–Meier survival and Cox regression analyses were performed to investigate the impact of *CYP24A1* DNA methylation on the prognosis of CRC patients. Prognostic risk scores were constructed for survival prediction. Immune infiltration analysis was also conducted. Our results showed that the hypermethylation of cg02712555 in tumor tissues (hazard ratio, 0.48; 95% confidence interval, 0.24–0.94; *p* = 0.032) and CpG site 41 in peripheral leukocytes (HR, 0.35; 95%CI, 0.14–0.84; *p* = 0.019) were both associated with decreased overall mortality in CRC patients. Prognostic risk scores showed robust predictive capabilities of these two CpG loci for the prognosis of CRC patients. *CYP24A1* hypermethylation was positively correlated with infiltration levels of activated CD4 + T cells, activated CD8 + T cells, activated B cells, activated dendritic cells, and macrophages. Taken together, our findings indicate that the methylation levels of specific CpG sites within the *CYP24A1* promoter region in blood leukocytes and tumors are potential prognostic and predictive markers for overall survival in CRC patients.

## 1. Introduction

Colorectal cancer (CRC) ranks as the third most common cancer and the second leading cause of cancer-related death globally [1]. In 2022, approximately 1.9 million new cases and 0.9 million deaths were reported worldwide [1]. Over the past two decades, the incidence and mortality of CRC have increased in China [2]. In 2022, CRC was the second most common cancer in China, with an estimated 517,100 new cases [2]. Despite improvements in survival rates for CRC patients in China over the past decade, the overall prognosis remains poor with a 5-year relative survival rate during 2012–2015 still below 60% [3].

The occurrence and progression of CRC are influenced by various factors. Accumulating studies have indicated that 1,25-dihydroxyvitamin D_3_ (1,25(OH)_2_D_3_) exerts antineoplastic effects by inhibiting the proliferation, invasion, and metastasis of various cancer cell types [4,5,6,7,8]. *CYP24A1* encodes 24-hydroxylase, a key enzyme that catabolizes 1,25(OH)_2_D_3_ into less active metabolites [9], thereby reducing 1,25(OH)_2_D_3_ levels and contributing to cancer development and progression. It has been reported that *CYP24A1* is upregulated in several cancer types, including CRC [10,11,12,13]. Furthermore, the increased expression of *CYP24A1* has been associated with the poorer survival of CRC patients [13].

*CYP24A1* has long CpG islands in its promoter region, and its expression can be regulated by DNA methylation [14]. DNA methylation, a crucial epigenetic modification, converts cytosine in DNA to 5-methylcytosine [15], regulating gene expression, particularly in promoter regions [16]. With growing evidence underscoring the significance of epigenetic alterations in CRC progression, the role of DNA methylation has garnered increasing attention. Aberrant DNA methylation changes have been recognized as crucial phenomena in CRC progression [17] and could serve as important prognostic markers. Multi-omics analysis of CRC has revealed an association between the hypermethylation of heparanase 2 (*HPSE2*) and the poor prognosis of CRC patients [18]. Previous research found that the methylation of the synaptic membrane exocytosis 2 (*RIMS2*) promoter had independent predictive value for overall survival in CRC patients [19]. Regarding the impact of *CYP24A1* DNA methylation on CRC, our previous study found that methylation in the promoter region of *CYP24A1* was inversely associated with CRC risk [20]. However, the prognostic value of *CYP24A1* DNA methylation in CRC patients has not yet been reported.

This study aimed to explore the prognostic value of *CYP24A1* DNA methylation in CRC patients. Considering that obtaining blood samples is non-invasive and more convenient compared to tissue samples, which could enhance the clinical application of potential prognostic predictors, blood samples were included in addition to tissue samples.

## 2. Materials and Methods

### 2.1. Sample Collection for the Blood Cohort

Patients with CRC treated at Sun Yat-sen University Cancer Center (Guangzhou, China) were recruited for this study from July 2010 to May 2021. The inclusion criteria required patients to be between 30 and 75 years old and to have received a histological diagnosis within the past 3 months at the time of recruitment. Patients were excluded if they had a history of other cancers or had undergone any preoperative therapy (radiotherapy or chemotherapy). As a result, a total of 101 patients were enrolled in the study. Healthy controls were recruited following the same inclusion criteria as the patients, except that they had no history of any cancers. A total of 101 healthy controls, frequency-matched with the patients by sex and age (within a 5-year interval), were enrolled during the same period. The healthy controls were inpatients admitted to the First Affiliated Hospital of Sun Yat-sen University, the Affiliated Eye Hospital of Sun Yat-sen University, and residents from the same community as the patients.

Overnight fasting peripheral blood samples were collected from participants on the morning of the second day after admission without any prior treatment. After collection, the blood samples were immediately centrifuged at 3000 rpm for 15 min at 4 °C. Leukocytes were then separated into EP tubes for DNA extraction, and all blood specimens were stored at −80 °C until further use. Information on patient characteristics, including age, sex, and TNM stage, was also collected. All patients and healthy controls provided written informed consent. This study was approved by the Ethics Committee of the School of Public Health, Sun Yat-sen University (approval number: 2019-105).

### 2.2. Cell Culture and DAC Treatment

The normal human colonic epithelial cell line CCD 841 CoN and CRC cell lines DLD1, Lovo, HCT116, and SW480 were obtained from the Colorectal Department Laboratory at Sun Yat-sen University Cancer Center. CCD 841 CoN cells were cultured in EMEM medium, HCT116 cells in McCoy’s 5A medium, and DLD1, Lovo, and SW480 cells in RPMI 1640 medium. All media were supplemented with 10% fetal bovine serum and 1% penicillin/streptomycin, and the cells were maintained at 37 °C in a humidified atmosphere containing 5% CO_2_. All reagents mentioned above were purchased from Gibco (Thermo Fisher Scientific, Waltham, MA, USA). To promote DNA demethylation, DLD1 and Lovo cells were treated with various concentrations of 5-aza-2′-deoxycytidine (DAC) (Sigma-Aldrich, St. Louis, MO, USA) at 4, 8, 16, and 32 μM for 48 h. Control groups were cultured with an equivalent amount of DMSO (Sigma-Aldrich, St. Louis, MO, USA) solvent.

### 2.3. RNA Extraction, Reverse Transcription and qRT-PCR

Total RNA was extracted from cultured cells using TRIzol reagent (Invitrogen, Carlsbad, CA, USA) following the manufacturer’s instructions. Reverse transcription was performed on 2 μg of RNA using the 5×All-in-One RT MasterMix kit (Applied Biological Materials, Richmond, BC, Canada). Real-time PCR was then performed using the KAPA SYBR^®^ FAST Universal Kit (KAPA Biosystems, Wilmington, MA, USA). All experiments were performed in triplicate, and the relative expression level of *CYP24A1* was normalized to *GAPDH* using the 2^−ΔΔCt^ method. The primers used for amplifications were as follows: *GAPDH*-Forward: TATCGTGATGCTAGTCCGATG, *GAPDH*-Reverse: TGCAGCTAGCTGCATCGATCGG, *CYP24A1*-Forward: AGCGATAATACGCCTCAGATGG, *CYP24A1*-Reverse: GATGGTGCTGACACAGGTGA.

### 2.4. Western Blot Analysis

Total protein was lysed using RIPA buffer (Thermo Fisher Scientific, Waltham, MA, USA). Protein lysates were separated by 10% sodium dodecyl sulfate polyacrylamide gel electrophoresis (SDS-PAGE) and transferred to PVDF membranes (Bio-Rad, Hercules, CA, USA). The membranes were blocked with 5% skim milk in tris-buffered saline with 0.05% Tween 20 (TBST) buffer for 1 h at room temperature, followed by incubation with primary antibodies (CYP24A1, 1:1000, Novus Biologicals, Centennial, CO, USA; GAPDH, 1:1000, Cell Signaling Technology, Danvers, MA, USA) overnight at 4 °C. After three washes with TBST, the membranes were incubated with corresponding horseradish peroxidase (HRP)-conjugated secondary antibody (1:1000, Cell Signaling Technology, Danvers, MA, USA). All experiments were performed in triplicate. Specific bands were visualized using ECL chromogenic substrate. Quantification was performed by densitometry using Image J 1.53c software (NIH, Bethesda, MD, USA), and the values were normalized to corresponding GAPDH control. Original western blots can be found at Supplementary Materials (Figure S1).

### 2.5. DNA Extraction and Bisulfite Conversion

Genomic DNA was isolated from leukocytes and cultured cells using the Genomic DNA Extraction Kit (TIANGEN Biotech, Beijing, China) according to the manufacturer’s instructions. The quality of the extracted DNA was assessed using NanoDrop ND-2000 (Thermo Fisher Scientific, Waltham, MA, USA) and agarose gel electrophoresis. Nanodrop ND-2000 was employed to determine the concentration and purity of DNA and agarose gel electrophoresis was used to evaluate the integrity of DNA, enabling eligible DNA samples exhibiting an OD ratio (A260/A280) within the range of 1.8–2.0 and clear main bands without significant dispersion or trailing. Following the manufacturer’s instructions, qualified DNA samples were then subjected to bisulfite conversion using the EZ DNA Methylation Gold™ Kit (Zymo Research Corporation, Irvine, CA, USA).

### 2.6. MethylTarget Sequencing and Methylation Data Processing

MethylTarget^TM^, a next-generation sequencing-based method for multiple targeted CpG islands methylation detection, was used to determine the DNA methylation levels of *CYP24A1* and was performed by Genesky BioTech (Shanghai, China). CpG islands were selected based on criteria of more than 200 bp in length, more than 50% GC content, and a CpG observation expectation ratio greater than 0.6. This selection process identified four candidate CpG islands containing 71 CpG sites for MethylTarget sequencing.

Based on DNA samples treated with bisulfite, the primers were designed and optimized for multiplex polymerase chain reaction (PCR) using Methylation Fast Target 4.1 software, and the optimized primers were combined into a multiplex PCR primer panel. PCR products were separated by agarose electrophoresis and purified using the Agarose Gel Extraction Kit (TIANGEN Biotech, Beijing, China). Libraries from different samples were quantified, pooled, and sequenced on the Illumina Hiseq platform (San Diego, CA, USA) in 2 × 150 bp paired-end mode, following the manufacturer’s protocol. After sequencing, Fast QC 0.11.5 software was used to assess the quality of data.

### 2.7. Data Sources for the Tissue Cohort

Preprocessed data from the Illumina HumanMethylation450 BeadChip, San Diego, CA, USA (450K array) on primary tumor tissues and normal tissues of CRC patients were collected for differential methylation analysis, including datasets GSE48684 and GSE193535 from the Gene Expression Omnibus (GEO) database.

Level 3 RNA-seq data (raw read counts) from primary tumor tissues and normal tissues were downloaded from the repository of colon adenocarcinoma (COAD) and rectal adenocarcinoma (READ) in the Cancer Genome Atlas (TCGA) database via the Genomic Data Commons (GDC) portal for differential expression analysis. Additionally, samples with both 450K array and RNA-seq data were selected for correlation analysis between DNA methylation and gene expression of *CYP24A1*. Patients with available 450K array data and clinical information were chosen for survival analysis.

### 2.8. Differential Methylation Analysis

In the blood cohort, CpG sites within the promoter region of *CYP24A1* were selected following the criteria of 2500 bp upstream to 1000 bp downstream of the transcription start site (TSS). Differential methylation analysis was first performed on promoter CpG sites individually between CRC patients and healthy controls. CpG sites with a *p*-value less than 0.05 were defined as significant CpG sites and selected for subsequent analyses. The methylation levels of identified significant CpG sites were then averaged to quantify the promoter methylation level of *CYP24A1*. Based on the averaged methylation level, differential methylation analysis was then performed on the promoter of *CYP24A1*.

In the GEO tissue cohort, the methylation level of each CpG site covered by the 450K array was represented as the beta-value (β), which was the ratio of the methylation intensity and the overall intensity and ranged from 0 to 1. The R package “Illumina HumanMethylation450kanno.ilm10b2.hg19” 0.6.1 was utilized to select CpG probes that were annotated to *CYP24A1* and located on the promoter-like regions (TSS1500, TSS200, 5′untranslated region (UTR), first exon) of the gene. Similarly, differential methylation analysis was initially performed on the selected promoter CpG sites individually between primary tumor tissues and normal tissues in the GSE48684 dataset comprising 64 primary tumor tissues and 24 normal tissues, and those with a *p*-value less than 0.05 were defined as significant CpG sites. Additionally, 450K array data of 54 primary tumor tissues and 54 matched adjacent normal tissues in the GSE193535 dataset were used for further identification of significant CpG sites. Only significant CpG sites identified in the GSE193535 dataset were considered for quantification of the promoter methylation level of *CYP24A1* and subsequent analyses. Differential methylation analysis was then performed on the promoter of *CYP24A1*.

### 2.9. Differential Expression Analysis and In Vitro Validation

Two subsets of RNA-seq data from the TCGA database were used for the differential expression analysis of *CYP24A1*. For normalization, the raw count data underwent log transformation using the formula log2(counts + 1). The first subset, consisting of 641 primary tumor tissues and 51 normal tissues, was used to evaluate the differential expression of *CYP24A1*. Subsequently, the second subset, including 50 primary tumor tissues and 50 matched adjacent normal tissues, was utilized to further identify the differential expression of *CYP24A1*.

To validate the differential expression of *CPY24A1*, additional in vitro experiments were conducted using CRC cell lines and a normal human colonic epithelial cell line, as detailed above.

### 2.10. Functional Enrichment Analysis for CYP24A1 and CYP24A1-Related Genes

Using the STRING tool, we identified 50 CYP24A1-binding proteins, with the following main parameters: minimum required interaction score [“Low confidence (0.150)”], meaning of network edges (“evidence”), max number of interactors to show (“no more than 50 interactors” in 1st shell), and active interaction sources (“experiments”). Additionally, the “Similar Gene Detection” module of GEPIA2 was used to obtain the top 100 genes correlated with *CYP24A1* expression across CRC tumor and normal tissues in the TCGA dataset. These 150 genes, along with *CYP24A1*, were subjected to Gene Ontology (GO) and Kyoto Encyclopedia of Genes and Genomes (KEGG) enrichment analyses using the “clusterprofiler” 4.10.0 R package. For GO enrichment analysis, *p*-values were adjusted for multiple comparisons using the Benjamini–Hochberg method, while for KEGG enrichment analysis, *p*-values were corrected using the false discovery rate (FDR) method. A *p*.adjust-value of less than 0.05 was considered statistically significant.

### 2.11. Correlation Analysis of DNA Methylation and Gene Expression and In Vitro Validation

Considering the potential role of significant CpG sites in gene regulation, Spearman correlation analysis was performed in the TCGA tissue cohort to evaluate the relationship between DNA methylation and gene expression of *CYP24A1* at both individual significant CpG site and promoter levels. Raw read counts were converted to transcripts per million (TPM) to represent gene expression levels. Significant CpG sites and promoters with Spearman correlation coefficients (*r*) less than −0.3 and *p*-values less than 0.05 were considered significant and were further analyzed. To validate the impact of DNA methylation on *CYP24A1* gene expression, in vitro experiments were conducted using two CRC cell lines with varying degrees of differentiation and treated with demethylation. Detailed information on these experiments is provided above.

### 2.12. Survival Analysis and Construction of Prognostic Risk Scores

In both the blood and TCGA tissue cohorts, Kaplan–Meier survival analysis and Cox regression analysis (both univariable and multivariable) were first performed using the R package “survival” 3.6-4 to explore the influence of DNA methylation of *CYP24A1* on the prognosis of CRC patients. To further elucidate the prognostics significance of *CYP24A1* DNA methylation, prognostic risk scores based on DNA methylation of specific CpG sites were then constructed. First of all, patients included in Cox regression analysis in both the blood and TCGA tissue cohorts were randomly divided into a training set and test set at the ratio of 0.7 and 0.3, respectively, using the “createDataPartition” function of R package “caret” 6.0-94. In the training sets, multivariable Cox regression analyses were performed, and prognostic risk scores were obtained using the “predict” function from the “stats” 4.3.2 R package. The multivariable Cox regression models in the training sets were then implemented in the test sets to calculate prognostic risk scores for the patients. Time-dependent receiver operating characteristics (ROC) analyses associated with 3- and 5-year survival were performed using the R package “timeROC” 0.4 and the area under the curve (AUC) values were calculated to assess the predictive power of the prognostic risk scores. In addition, Kaplan–Meier analysis was employed to evaluate the ability of the prognostic risk scores to distinguish overall survival of CRC patients.

### 2.13. Immune Infiltration Analysis

Immune infiltration analysis was conducted using the Tumor–Immune System Interactions Database (TISIDB) to evaluate the relationship of *CYP24A1* methylation and the abundance of various tumor-infiltrating lymphocytes (TILs), including activated CD4 + T cells, activated CD8 + T cells, activated B cells, activated dendritic cells, macrophages, and neutrophils. Spearman correlation analysis was performed, and a *p*-value of less than 0.05 was considered statistically significant.

### 2.14. Statistical Analysis

The Mann–Whitney U test was employed for unpaired samples, and the Wilcoxon signed-rank test was employed for paired samples to compare differences in methylation and expression levels between two groups in the blood and tissue cohorts. The hazard ratio (HR) and 95% confidence interval (CI) were assessed using univariable and multivariable Cox regression analyses. One-way ANOVA was conducted to compare expression levels among more than two groups in qRT-PCR and Western blot, followed by Dunnett’s test for pairwise comparison between treatment groups and the control group. The log-rank test was used to determine significant differences between the two groups in the Kaplan–Meier survival analysis. All analyses were conducted using R version 4.4.1, except for the qRT-PCR and Western blot results, which were analyzed using GraphPad Prism 10 (GraphPad Software, La Jolla, CA, USA). All statistical tests were two-sided, with a significance level set at *p* < 0.05.

## 3. Results

### 3.1. Identification of Significant Promoter CpG Sites in the Blood Cohort and GEO Tissue Cohort

In the blood cohort, 7 CpG sites outside the promoter region of *CYP24A1* were excluded and 64 promoter CpG sites were retained (as detailed in Table S1). In total, 4 out of 64 promoter CpG sites were identified as significant CpG sites between CRC patients and healthy controls (*p* < 0.05). This included three hypomethylated sites (CpG site 102, CpG site 200, and CpG site 41) and one hypermethylated site (CpG site 53). These four significant CpG sites were subsequently included in further analyses. To quantify the promoter methylation level of *CYP24A1*, two strategies were employed, as the four significant CpG sites showed different methylation trends in CRC patients. When promoter methylation level was quantified by averaging the methylation levels of only the three hypomethylated CpG sites (defined as Promoter 1), a significant difference was observed between CRC patients and healthy controls (*p* < 0.05). However, this significance was not retained when the methylation levels of all four significant CpG sites were averaged (defined as Promoter 2). Hence, the values of Promoter 1 were used to represent the promoter methylation levels of *CYP24A1* in subsequent analyses (Table 1).

**Figure 1:**
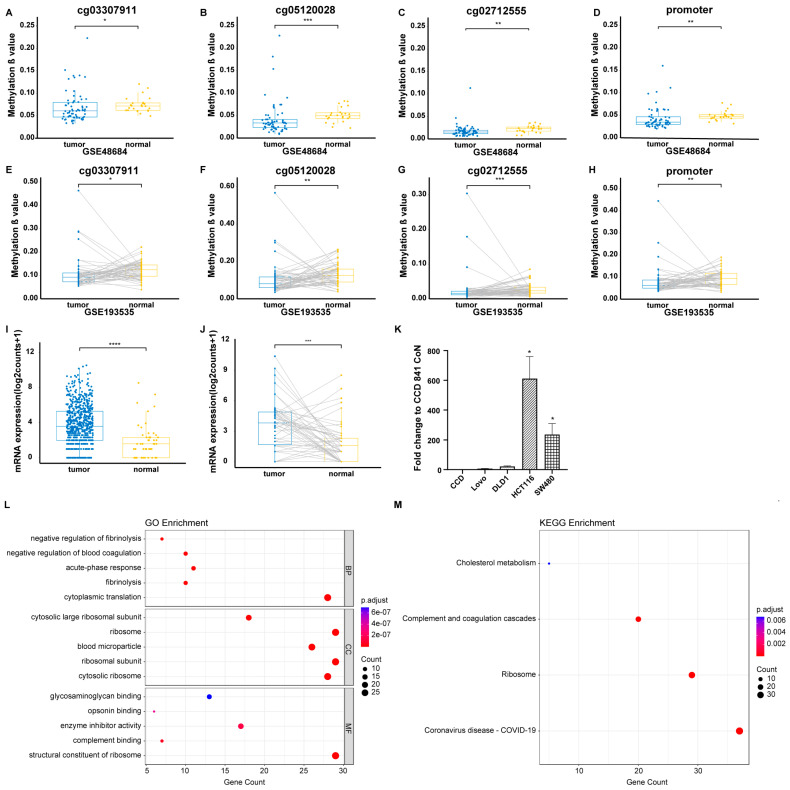
Differential methylation and expression analyses of *CYP24A1*. Significant CpG sites/promoter between 64 primary tumor tissues and 24 normal tissues in the GSE48684 dataset (**A**–**D**) and between 54 primary tumor tissues and 54 matched adjacent normal tissues in the GSE193535 dataset (**E**–**H**). *CYP24A1* was differentially expressed between unpaired tumor tissues (n = 641) and normal tissues (n = 51) (**I**), and between paired tumor tissues (n = 50) and adjacent normal tissues (n = 50) (**J**) in the TCGA tissue cohort with higher expression level in tumor tissues compared to normal tissues. (**K**) Significant upregulation in expression level of *CYP24A1* was observed in both HCT116 and SW480 CRC cell lines compared to the normal human colonic epithelial cell line CCD 841 CoN. Mean ± SEM of N = 3 independent replicates is shown. (**L**) GO enrichment analysis result (top 5 terms of each subtype) for *CYP24A1* and the related genes. (**M**) KEGG pathway enrichment result for *CYP24A1* and the related genes. * *p* < 0.05, ** *p* < 0.01, *** *p* < 0.001, **** *p* < 0.0001; SEM, standard error of the mean; GEO, Gene Expression Omnibus; TCGA, The Cancer Genome Atlas; CRC, colorectal cancer; MF, molecular function; CC, cellular component; BP, biological process.

In the GEO tissue cohort, three significant CpG sites (cg03307911, cg05120028, and cg02712555), which were all hypomethylated in tumor samples, were screened out (Figure 1A–C) among seven promoter CpG sites (*p* < 0.05). These three significant CpG sites were further identified (Figure 1E–G) in the GSE193535 dataset and were retained for subsequent analyses. As shown in Figure 1D,H, a significant difference in promoter methylation level of *CYP24A1*, which was quantified by averaging the methylation levels of all three significant CpG sites, was observed between tumor tissues and adjacent normal tissues in both GSE48684 and GSE193535 datasets (*p* < 0.05).

### 3.2. Identification of Differential Expression of CYP24A1 in the TCGA Tissue Cohort and Validation Through In Vitro Experiments

As shown in Figure 1I,J, *CYP24A1* was significantly overexpressed in tumor tissues compared to normal tissues in both the dataset with 692 unpaired samples and the dataset with 100 paired samples from the TCGA database (*p* < 0.05). This upregulation of *CYP24A1* was further validated through in vitro experiments. Among all detected CRC cell lines, a higher expression level of *CYP24A1* was confirmed in HCT116 and SW480 cells in comparison with CCD 841 CoN (*p* < 0.05) (Figure 1K).

### 3.3. GO and KEGG Enrichment Analyses of CYP24A1 and CYP24A1-Related Genes

GO and KEGG enrichment analyses were performed to investigate the molecular mechanisms by which *CYP24A1* contributes to CRC tumorigenesis and progression. Using STRING and GEPIA2, a list of *CYP24A1* and 150 *CYP24A1*-related genes (as detailed in Tables S2 and S3) was compiled for functional enrichment analysis. GO analysis showed that most of these genes were significantly associated with cytoplasmic translation in the biological process (BP) category, cytosolic ribosome in cellular component (CC) category, and the structural constituent of ribosome in the molecular function (MF) category (Figure 1L). KEGG analysis (Figure 1M) suggested that “Ribosome”, “Complement and coagulation cascades”, and “Cholesterol metabolism” pathways might be involved in the effect of *CYP24A1* on CRC development and progression.

### 3.4. Correlation Analysis of DNA Methylation and Gene Expression of CYP24A1 in the TCGA Tissue Cohort and Validation Through In Vitro Experiments

**Figure 2:**
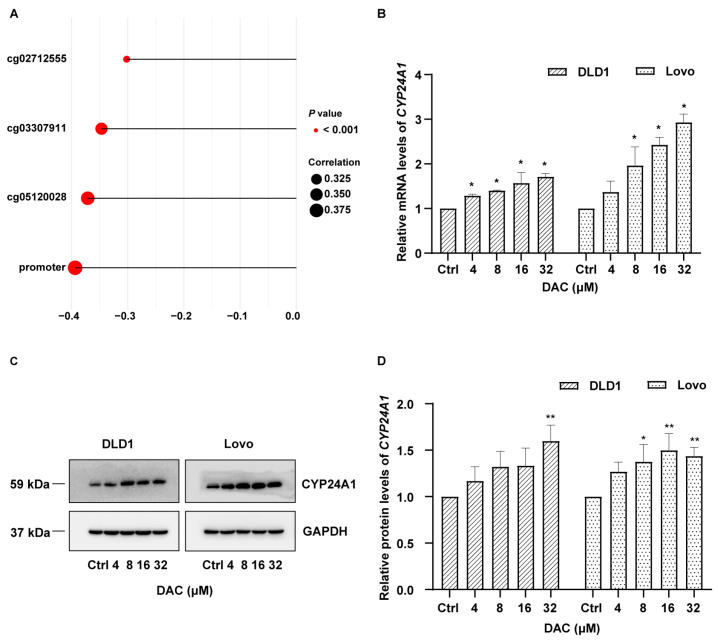
Relationship between DNA methylation and gene expression of *CYP24A1*. (**A**) Spearman correlation analysis of DNA methylation of significant CpG sites/promoter and gene expression of *CYP24A1* in the TCGA tissue cohort. (**B**) The changes in mRNA levels of *CYP24A1* before and after DAC treatment at various concentrations in DLD1 and Lovo CRC cell lines. Mean ± SEM of N = 3 independent replicates is shown. (**C**,**D**) The changes in protein levels of *CYP24A1* before and after DAC treatment at various concentrations in DLD1 and Lovo CRC cell lines. Mean ± SEM of N = 3 independent replicates is shown. * *p* < 0.05, ** *p* < 0.01; DAC, 5-aza-2′-deoxycytidine; SEM, standard error of the mean; TCGA, The Cancer Genome Atlas; CRC, colorectal cancer.

A total of 402 tissue samples with both 450K array and RNA-seq data from the TCGA database were included for the correlation analysis of DNA methylation and gene expression of *CYP24A1*. The methylation levels of the three significant CpG sites identified in the GEO tissue cohort were all negatively correlated with the gene expression of *CYP24A1* (*p* < 0.05, *r* < −0.3). Similarly, the promoter methylation level was also negatively correlated with *CYP24A1* gene expression (Figure 2A).

Among the four CRC cell lines, Lovo was moderately to highly differentiated, while DLD1, HCT116, and SW480 were all poorly differentiated. To cover different differentiation levels, DLD1 and Lovo were chosen for DAC treatments. After DAC treatment at the highest concentration (32 μM), the methylation level of *CYP24A1* decreased from 0.51 to 0.45 in the DLD1 cell line and from 0.13 to 0.10 in the Lovo cell line. In the DLD1 cell line, significantly increased mRNA levels of *CYP24A1* were observed after treatment with various concentrations of DAC. In the Lovo cell line, similar significant upregulations in *CYP24A1* mRNA levels were obtained after DAC treatment, except at the concentration of 4 μM (*p* < 0.05) (Figure 2B). Similar to the mRNA results, the protein levels of *CYP24A1* increased after DAC treatment in both DLD1 and Lovo cell lines (Figure 2C,D).

### 3.5. Clinical Characteristics of CRC Patients Included in the Survival Analysis

In the blood cohort, a total of 101 CRC patients were included in the Kaplan–Meier survival analysis. However, two patients were excluded from the Cox regression analysis due to missing TNM stage information. Overall, patients in the blood cohort had an average age of 58 years, a higher portion of males than females, and more patients with stage III/IV disease compared to those with stage I/II.

In the TCGA tissue cohort, 380 patients were included in the Kaplan–Meier survival analysis. However, eighteen patients without TNM stage information were excluded, resulting in a final sample size of 362 for the Cox regression analysis. Unlike the blood cohort, patients in the TCGA cohort had an older average age of 65 years and a lower portion of patients with stage III/IV disease compared to those with stage I/II. Detailed patient characteristics are summarized in Table 2.

### 3.6. Prognostic Value of DNA Methylation of CYP24A1 in CRC

Using the “surv_cutpoint” function of the “survminer” 0.4.9 R package, cut-off values of DNA methylation levels in patients were determined, and patients were then divided into hypomethylation and hypermethylation groups based on the cut-off values. In the blood cohort, the thresholds for individual methylation levels of the four significant CpG sites (CpG site 102, CpG site 200, CpG site 41, and CpG site 53) and the promoter methylation level were 0.026, 0.004, 0.016, 0.045, and 0.022, respectively. In the TCGA tissue cohort, the thresholds for individual methylation levels of the three significant CpG sites (cg03307911, cg05120028, and cg02712555) and the promoter methylation level were 0.051, 0.020, 0.015, and 0.032, respectively.

**Figure 3:**
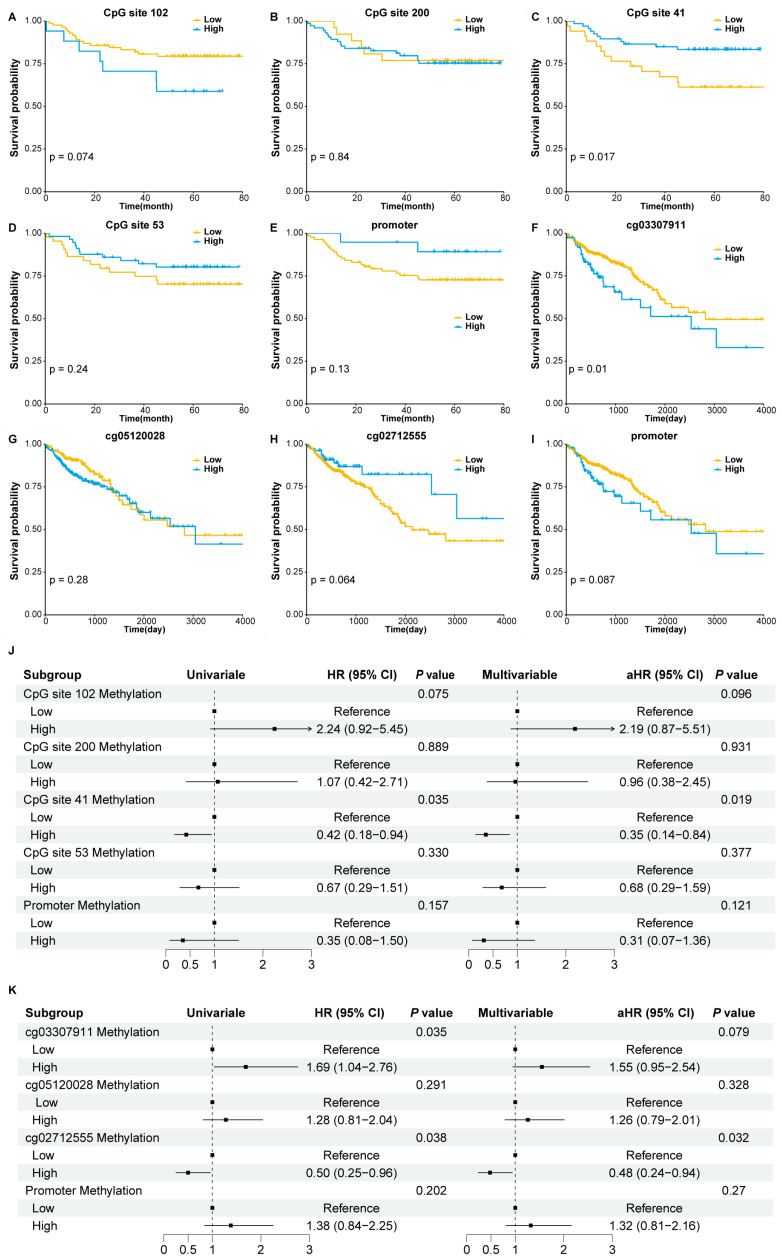
Survival analysis of methylation levels of significant CpG sites/promoter and overall survival of CRC patients. (**A**–**E**) The Kaplan–Meier curves of CRC patients with low versus high methylation levels of significant CpG sites/promoter in the blood cohort. (**F**–**I**) The Kaplan–Meier curves of CRC patients with low versus high methylation levels of significant CpG sites/promoter in the TCGA tissue cohort. Cox regression analysis of significant CpG sites/promoter methylation levels and CRC prognosis in the blood cohort (**J**) and TCGA tissue cohort (**K**). Multivariable Cox regression analysis adjusted for age, sex, and TNM stage. TCGA, The Cancer Genome Atlas; CRC, colorectal cancer.

Kaplan–Meier survival curves indicated that patients with higher methylation levels of CpG site 41 had significantly better survival (*p* < 0.05) (Figure 3C). Similarly, patients with higher methylation levels of cg02712555 exhibited better survival, though not statistically significant (Figure 3H). In contrast, patients with higher methylation levels of cg03307911 had significantly poorer survival (*p* < 0.05) (Figure 3F). No significant differences were observed between the two groups for other CpG sites or the promoter in both the blood cohort (Figure 3A,B,D,E) and TCGA tissue cohort (Figure 3G,I).

Consistent with the Kaplan–Meier survival analysis, the univariable Cox regression analysis revealed that the hypermethylation of CpG site 41 was significantly associated with a decreased risk of all-cause mortality in CRC patients, with an HR of 0.42 (95%CI, 0.18–0.94; *p* = 0.035). This association remained significant after adjusting for age, sex, and TNM stage in the multivariable analysis, with an HR of 0.35 (95%CI, 0.14–0.84; *p* = 0.019) (Figure 3J). As shown in Figure 3K, the methylation level of cg03307911 was positively associated with the overall mortality of CRC patients in the univariable Cox regression analysis (HR, 1.69; 95%CI, 1.04–2.76; *p* = 0.035), but not in the multivariable analysis (HR, 1.55; 95%CI, 0.95–2.54; *p* = 0.079). Conversely, cg02712555, which did not significantly differentiate survival in the Kaplan–Meier analysis, was inversely associated with the overall mortality of CRC patients in both the univariable and multivariable Cox regression analyses, with HRs of 0.50 (95%CI, 0.25–0.96; *p* = 0.038) and 0.48 (95%CI, 0.24–0.94; *p* = 0.032), respectively. Taken together, these findings indicated that the methylation level of CpG site 41 in the blood cohort and cg02712555 in the TCGA tissue cohort were significant predictors of overall survival in CRC patients, supporting their inclusion in subsequent prognostic risk scores construction.

### 3.7. Predictive Performances of Prognostic Risk Scores for Overall Survival in CRC Patients

**Figure 4:**
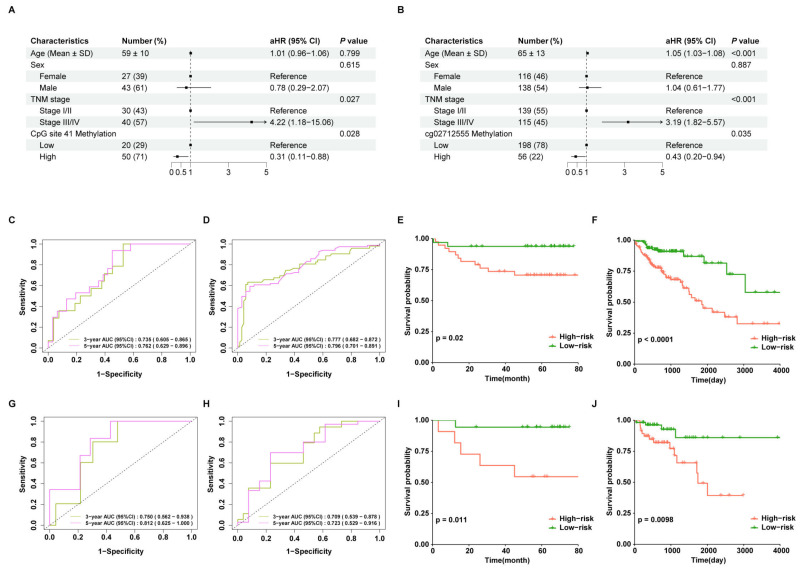
Predictive performances of the prognostic risk scores for overall survival in CRC patients. The forest plots for multivariable Cox regression models predicting overall survival of CRC patients in the training sets of blood cohort (**A**) and TCGA tissue cohort (**B**). Time-dependent ROC curve analysis of prognostic risk scores for predicting 3- and 5-year survival of CRC patients in the training sets of blood cohort (**C**) and TCGA tissue cohort (**D**). The Kaplan–Meier curves of CRC patients with low versus high prognostic risk scores and overall survival in the training sets of blood cohort (**E**) and TCGA tissue cohort (**F**). Time-dependent ROC curve analysis of the prognostic risk scores for predicting 3- and 5-year survival of CRC patients in the test sets of blood cohort (**G**) and TCGA tissue cohort (**H**). The Kaplan–Meier curves of CRC patients with low versus high prognostic risk scores and overall survival in the test sets of blood cohort (**I**) and TCGA tissue cohort (**J**). TCGA, The Cancer Genome Atlas; CRC, colorectal cancer; ROC, receiver operating characteristics.

Figure 4A,B illustrates multivariable Cox regression models constructed based on age, sex, TNM stage, and the methylation level of CpG site 41 in the training set of the blood cohort, and based on age, sex, TNM stage, and the methylation level of cg02712555 in the training set of the TCGA tissue cohort, with sample sizes of 70 and 254, respectively. Time-dependent ROC curves (Figure 4C,D,G,H) demonstrated the predictive performance of the prognostic risk scores. In the blood cohort, the AUC values for predicting the 3- and 5-year survival of CRC patients were 0.735 (95%CI, 0.605–0.865) and 0.762 (95%CI, 0.629–0.896) in the training set and 0.750 (95%CI: 0.562–0.938) and 0.812 (95%CI: 0.625–1.000) in the test set, respectively. Similarly, in the TCGA tissue cohort, the AUC values for predicting 3- and 5-year survival were 0.777 (95%CI: 0.682–0.872) and 0.796 (95%CI: 0.701–0.891) in the training set and 0.709 (95%CI: 0.539–0.878) and 0.723 (95%CI: 0.529–0.916) in the test set, respectively.

Patients in both the training and test sets of both cohorts were divided into low-risk and high-risk groups based on the mean prognostic risk scores for the Kaplan–Meier survival analysis. The Kaplan–Meier survival curves revealed that the prognostic risk scores effectively differentiated overall survival among CRC patients, not only in the training sets but also in the test sets of both cohorts (Figure 4E,F,I,J). Patients with lower prognostic risk scores exhibited significantly better survival outcomes (*p* < 0.05).

### 3.8. Correlation of CYP24A1 Methylation with Immune Infiltration in CRC

**Figure 5:**
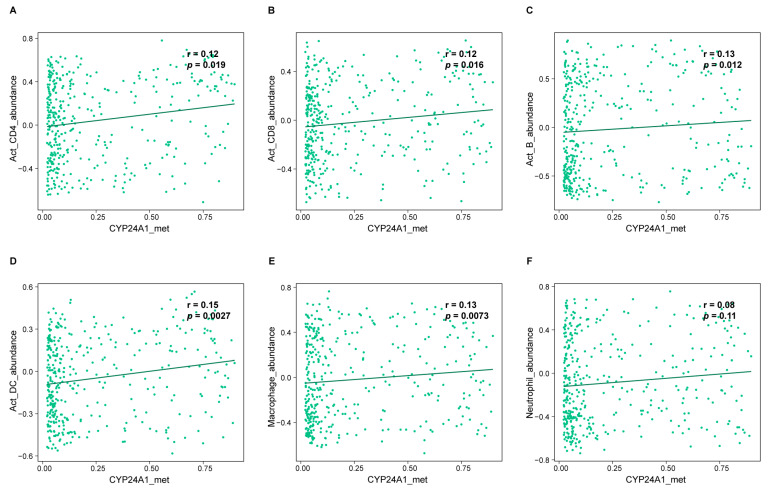
Correlation analysis of *CYP24A1* methylation with immune infiltration levels in CRC. DNA methylation of *CYP24A1* was significantly positively correlated with infiltration levels of activated CD4 + T cells (**A**), activated CD8 + T cells (**B**), activated B cells (**C**), activated dendritic cells (**D**), macrophages (**E**), and was not significantly correlated with infiltration levels of neutrophils (**F**) in TISIDB (n = 396). TISIDB, Tumor–Immune System Interactions Database.

As shown in Figure 5A–E, the DNA methylation of *CYP24A1* was found to be significantly positively correlated with the infiltration levels of activated CD4 + T cells, activated CD8 + T cells, activated B cells, activated dendritic cells, and macrophages. However, no significant correlation was observed between *CYP24A1* DNA methylation and the infiltration level of neutrophils (Figure 5F).

## 4. Discussion

The current study is the first to investigate the association between the DNA methylation of *CYP24A1* and the prognosis of CRC patients. Our results showed that the hypermethylation of cg02712555 in tumor tissues was associated with better survival in CRC patients. Similarly, the hypermethylation of CpG site 41 in peripheral leukocytes was associated with better survival outcomes in CRC patients. Furthermore, prognostic risk scores constructed based on these two CpG loci in blood and TCGA tissue cohorts exhibited robust predictive capabilities for the prognosis of CRC patients. Our results also revealed a positive correlation between *CYP24A1* methylation and immune infiltration in CRC.

In the present study, we found that all three significant CpG sites in the GEO tissue cohort were hypomethylated. The promoter methylation level, quantified based on these three significant CpG sites, was lower in tumor tissues compared to normal tissues, suggesting promoter hypomethylation of *CYP24A1*. In line with our results, a previous study from Austria reported that the methylation level of two regions within the promoter of *CYP24A1* was lower in tumor tissues than in adjacent normal mucosa, although this difference was not statistically significant [21].

Our study found that *CYP24A1* was overexpressed in CRC tissues compared to normal tissues, consistent with previous reports [13,22]. Furthermore, our in vitro experiments confirmed that the mRNA levels of *CYP24A1* were upregulated in several CRC cell lines compared to the normal human colonic epithelial cell line. By inactivating 1,25(OH)_2_D_3_, overexpression of *CYP24A1* limited biological activities of 1,25(OH)_2_D_3_, potentially reducing its effectiveness in inhibiting the proliferation and motility of cancer cells [23]. It has been reported that inhibiting *CYP24A1* expression enhances the antitumor effect of 1,25(OH)_2_D_3_ in various cancer cell types, including CRC cells [23,24,25]. Furthermore, studies have shown that increased expression of *CYP24A1* was associated with the poor prognosis of patients with different cancer types, including CRC [13,26,27]. Both our GO and KEGG results showed that *CYP24A1* might be associated with ribosomal function. Previous studies have revealed that the dysregulation of ribosome biogenesis and function was related to multiple caner types, including CRC [28,29,30]. These enrichment analyses provided insights into the potential molecular mechanisms by which *CYP24A1* influences CRC tumorigenesis and progression.

Previous studies have observed that the expression of *CYP24A1* is partly regulated by DNA methylation in both lung [31] and prostate adenocarcinomas [32]. Considering the potential role of DNA methylation in regulating *CYP24A1* expression in CRC, we investigated the correlation between promoter DNA methylation of *CYP24A1* and its gene expression and validated it in vitro. Our results showed that the promoter hypomethylation of *CYP24A1* was correlated with the upregulation of *CYP24A1* mRNA levels in the TCGA tissue cohort. In vitro experiments on CRC cell lines showed increases in *CYP24A1* mRNA levels and decreases in DNA methylation levels following DAC treatment, further validating the vital role of DNA methylation in the regulation of *CYP24A1* expression. However, a previous study found no correlation between *CYP24A1* mRNA levels and its promoter DNA methylation status in CRC tissues [33], which contrasts with our findings. This discrepancy may be due to differences in the CpG sites included in the quantification of *CYP24A1* promoter methylation level. Transcription is often regulated by the methylation of specific CpG loci within a promoter rather than the entire promoter region [34]. Our study performed differential methylation analysis on promoter CpG sites and included significant CpG sites between CRC tumors and normal tissues in the quantification of *CYP24A1* promoter methylation levels, whereas the previous study [33] included all tested promoter CpG sites in their quantification.

To our knowledge, no study has reported the association between the DNA methylation of *CYP24A1* and the prognosis of CRC patients. Our study found that the methylation level of cg02712555 was negatively associated with the overall mortality of CRC patients, after adjusting for potential confounders in the TCGA tissue cohort. Additionally, the prognostic risk scores demonstrated good predictive performance for CRC survival, suggesting the prognostic value of *CYP24A1* DNA methylation in CRC. Unlike individual CpG loci, no associations were observed between the overall promoter methylation level of *CYP24A1* and CRC prognosis, highlighting the importance of specific location when evaluating DNA methylation biomarkers in cancer, as emphasized in a previous study [35].

Compared to tissue biopsies, blood biopsies offer a non-invasive alternative that does not risk tumor spread and can be performed repeatedly. Tumors have been reported to induce epigenetic alterations in circulating leukocytes, resulting in corresponding changes [36]. DNA methylation in peripheral blood cells can reflect related methylation information of the tumor [35,37]. Our results in the blood cohort showed that the promoter hypomethylation of *CYP24A1* was also present in the leukocytes of CRC patients compared to healthy controls, which was consistent with findings in the GEO tissue cohort. Additionally, DNA methylation in peripheral blood mononuclear cells (PBMCs) has been reported to be related to the occurrence and progression of several cancer types [38,39,40]. Notably, Ganapathi et al. found that increased *FOXP3* methylation in PBMCs was associated with poorer survival in CRC patients [37], unveiling the prognostic potential of blood-based DNA methylation in CRC. In our blood cohort, CRC patients with higher methylation levels of CpG site 41 had better survival compared to those with lower methylation levels, and the prognostic risk scores demonstrated good predictive performance for CRC survival, mirroring our findings in the TCGA tissue cohort. Overall, these results suggest broad application prospects for the blood-based DNA methylation of *CYP24A1*, particularly at specific loci, as prognostic and predictive markers in CRC patients.

Tumor-infiltrating immune cells, as prominent components of the tumor microenvironment, are closely related to cancer prognosis [41,42]. Studies have shown that tumor-infiltrating lymphocytes can inhibit tumor growth and are associated with improved outcomes in CRC patients [43,44]. Given our finding that *CYP24A1* hypermethylation was associated with better prognosis in CRC, we hypothesize that *CYP24A1* methylation is positively correlated with immune infiltration. Supporting this hypothesis, our immune infiltration analysis revealed a positive correlation between *CYP24A1* methylation and the levels of TILs, suggesting a potential immune-related mechanism by which *CYP24A1* methylation influences CRC patient prognosis.

Some limitations of our study should be acknowledged. First, we did not test all CpG sites in the promoter region of *CYP24A1*, which may result in partial conclusions. However, it is known that aberrant DNA methylation related to gene expression abnormities and cancer progression occurs more frequently in CpG islands or specific CpG loci within promoter regions, rather than the entire promoter region [34,45]. In our study, MethylTarget sequencing was used to detect multiple CpG islands within the promoter region of *CYP24A1*, accurately calculating the methylation level of each CpG site through high-depth sequencing data. Second, due to the difficulty of obtaining tissue samples, we used Illumina HumanMethylation450 BeadChip data from public databases. Although the 450K array covers most CpG sites in the human genome, it includes only a few CpG sites specific to *CYP24A1*, resulting in the non-overlap of significant CpG sites in the blood and tissue cohorts. Specifically, CpG site 41 in our blood cohort is located 431 bp downstream of the TSS of *CYP24A1*, while cg02712555 in the tissue cohort is located 208 bp upstream of the TSS of *CYP24A1*. Both sites are within the same regulatory region—the promoter region—and their DNA methylation may collectively influence the regulation of gene expression and contribute to CRC progression. However, the discrepancy between the CpG sites in the promoter region of *CYP24A1* in tissue samples and those in blood samples limited our ability to conduct an in-depth study based on the same sites across different sample types. Further studies are needed to explore whether the same prognostic hallmarks exist in both sample types.

## 5. Conclusions

In summary, our study identified a novel association between *CYP24A1* DNA methylation and the prognosis of CRC patients, which has not been previously reported. Specifically, higher methylation levels of cg02712555 in tumor tissues and CpG site 41 in the blood leukocytes were both associated with lower overall mortality in CRC patients. The prognostic risk scores demonstrated that CpG site 41 methylation in the blood cohort and cg02712555 methylation in the TCGA tissue cohort were strong predictors of CRC prognosis. Additionally, *CYP24A1* methylation was positively correlated with infiltration levels of TILs. These findings highlight the potential of DNA methylation at specific CpG sites within the *CYP24A1* promoter as prognostic markers for CRC, though further studies are necessary to confirm these results.

## Figures and Tables

**Table 1:** Differential methylation of *CYP24A1* between CRC patients and healthy controls in the blood cohort.

CpG Site/Promoter	Genome Position (GRCh38/hg38)	Distance to the TSS ^1^	Patients (n = 101) Mean ± SD	Controls (n = 101) Mean ± SD	*p*
102	chr20:54174368	−382	0.018 ± 0.011	0.021 ± 0.011	0.029
200	chr20:54174270	−284	0.011 ± 0.009	0.014 ± 0.009	0.007
41	chr20:54173555	431	0.021 ± 0.008	0.023 ± 0.007	0.046
53	chr20:54172987	999	0.050 ± 0.024	0.044 ± 0.011	0.025
Promoter 1	-	-	0.017 ± 0.006	0.019 ± 0.005	<0.001
Promoter 2	-	-	0.025 ± 0.008	0.025 ± 0.006	0.322

^1^ TSS, transcription start site.

**Table 2:** Clinical characteristics of patients included in the survival analysis.

Characteristics	Blood Cohort Number (%)		TCGA Cohort Number (%)	
	K-M Survival Analysis	Cox Regression Analysis ^1^	K-M Survival Analysis	Cox Regression Analysis ^2^
Overall	101	99	380	362
Age (years) (Mean ± SD)	58 ± 10	58 ± 10	65 ± 13	65 ± 13
Sex				
Male	58 (57)	58 (59)	205 (54)	194 (54)
Female	43 (43)	41 (41)	175 (46)	168 (46)
TNM stage				
Stage I/II	41 (41)	41 (41)	194 (51)	194 (54)
Stage III/IV	58 (57)	58 (59)	168 (44)	168 (46)
Not reported	2 (2)	/	18 (5)	/

^1^ Two patients were excluded from the Cox regression analysis in the blood cohort due to missing TNM stage information. ^2^ Eighteen patients were excluded from the Cox regression analysis in the TCGA tissue cohort due to missing TNM stage information.

## Data Availability

The datasets generated and/or analyzed during the current study are available in the GEO repository (https://www.ncbi.nlm.nih.gov/geo/, accessed on 28 April 2024), Genomic Data Commons Data portal (https://portal.gdc.cancer.gov/, accessed on 16 April 2024) and the corresponding author on reasonable request.

## Supplementary Materials

The following supporting information can be downloaded at: https://www.mdpi.com/article/10.3390/biom15010104/s1, Table S1: The information of 64 CpG sites within the promoter region of *CYP24A1* in the blood cohort; Table S2: The information of top 100 expression-correlated genes of *CYP24A1* from GEPIA2; Table S3: The information of 50 CYP24A1-binding proteins from STRING; Figure S1: The original western blots.
