# Supplementary material for: CYP24A1 DNA Methylation in Colorectal Cancer as Potential Prognostic and Predictive Markers
Source: Biomolecules. 2025 Jan 10;15(1):104. doi: 10.3390/biom15010104 (PMC11763947; doi:10.3390/biom15010104)
Supplement: Supplementary file 1 [file biomolecules-15-00104-s001.zip › biomolecules-3320003 -supplementary materials-update.pdf]

Table S1. The information of 64 CpG sites within the promoter region of *CYP24A1* in the blood cohort

CpG site	Genome position (GRCh38/hg38)	Distance to the TSS[1]	Patients (n=101) Mean ± SD	Controls (n=101) Mean ± SD	*P*
102	chr20:54174368	-382	0.018±0.011	0.021±0.011	0.029
200	chr20:54174270	-284	0.011±0.009	0.014±0.009	0.007
41	chr20:54173555	431	0.021±0.008	0.023±0.007	0.046
53	chr20:54172987	999	0.050±0.024	0.044±0.011	0.025
23	chr20:54174447	-461	0.010±0.009	0.009±0.006	0.943
30	chr20:54174440	-454	0.012±0.009	0.012±0.009	0.488
34	chr20:54174436	-450	0.012±0.010	0.010±0.006	0.460
43	chr20:54174427	-441	0.014±0.009	0.014±0.008	0.860
58	chr20:54174412	-426	0.013±0.009	0.011±0.008	0.136
61	chr20:54174409	-423	0.019±0.011	0.020±0.010	0.852
79	chr20:54174391	-405	0.025±0.014	0.024±0.011	0.797
85	chr20:54174385	-399	0.013±0.010	0.012±0.008	0.575
94	chr20:54174376	-390	0.013±0.009	0.013±0.008	0.877
98	chr20:54174372	-386	0.024±0.015	0.026±0.013	0.179
108	chr20:54174362	-376	0.019±0.013	0.019±0.011	0.755
110	chr20:54174360	-374	0.026±0.015	0.027±0.014	0.333
122	chr20:54174348	-362	0.018±0.012	0.017±0.011	0.513
125	chr20:54174345	-359	0.017±0.011	0.017±0.009	0.715
129	chr20:54174341	-355	0.014±0.010	0.012±0.008	0.322
138	chr20:54174332	-346	0.008±0.007	0.008±0.006	0.309
142	chr20:54174328	-342	0.016±0.011	0.017±0.011	0.439
145	chr20:54174325	-339	0.016±0.010	0.018±0.011	0.301
151	chr20:54174319	-333	0.011±0.009	0.012±0.007	0.148
162	chr20:54174308	-322	0.016±0.010	0.015±0.008	0.791
165	chr20:54174305	-319	0.029±0.015	0.032±0.014	0.128
175	chr20:54174295	-309	0.011±0.009	0.013±0.009	0.114
183	chr20:54174287	-301	0.019±0.011	0.017±0.008	0.368
185	chr20:54174285	-299	0.011±0.010	0.010±0.007	0.991
189	chr20:54174281	-295	0.015±0.012	0.014±0.009	0.587
210	chr20:54174260	-274	0.010±0.008	0.012±0.008	0.219
217	chr20:54174253	-267	0.013±0.009	0.014±0.009	0.557
45	chr20:54173551	435	0.023±0.009	0.023±0.008	0.895

52	chr20:54173544	442	0.028±0.010	0.029±0.009	0.299
71	chr20:54173525	461	0.026±0.010	0.026±0.010	0.795
78	chr20:54173518	468	0.017±0.006	0.017±0.007	0.585
90	chr20:54173506	480	0.014±0.005	0.014±0.005	0.162
108	chr20:54173488	498	0.026±0.011	0.028±0.010	0.179
111	chr20:54173485	501	0.023±0.011	0.024±0.010	0.625
117	chr20:54173479	507	0.019±0.008	0.019±0.007	0.301
129	chr20:54173467	519	0.023±0.008	0.023±0.007	0.292
131	chr20:54173465	521	0.012±0.006	0.012±0.005	0.688
150	chr20:54173446	540	0.045±0.017	0.042±0.012	0.116
166	chr20:54173430	556	0.048±0.013	0.047±0.013	0.845
178	chr20:54173418	568	0.027±0.011	0.026±0.009	0.912
180	chr20:54173416	570	0.026±0.012	0.027±0.009	0.447
184	chr20:54173412	574	0.040±0.013	0.040±0.012	0.968
192	chr20:54173404	582	0.019±0.009	0.019±0.007	0.585
20	chr20:54173157	829	0.028±0.012	0.026±0.009	0.747
25	chr20:54173152	834	0.029±0.014	0.029±0.010	0.348
31	chr20:54173146	840	0.022±0.011	0.022±0.007	0.716
34	chr20:54173143	843	0.050±0.016	0.050±0.015	0.661
43	chr20:54173134	852	0.023±0.010	0.023±0.009	0.473
45	chr20:54173132	854	0.023±0.010	0.022±0.007	0.929
54	chr20:54173123	863	0.037±0.014	0.035±0.013	0.601
56	chr20:54173121	865	0.024±0.012	0.023±0.007	0.866
65	chr20:54173112	874	0.028±0.011	0.027±0.010	0.421
69	chr20:54173108	878	0.028±0.011	0.027±0.009	0.751
111	chr20:54173066	920	0.065±0.019	0.064±0.026	0.372
136	chr20:54173041	945	0.057±0.018	0.058±0.016	0.527
32	chr20:54173008	978	0.039±0.013	0.040±0.011	0.640
40	chr20:54173000	986	0.037±0.013	0.036±0.012	0.946
45	chr20:54172995	991	0.038±0.015	0.037±0.011	0.659
51	chr20:54172989	997	0.041±0.017	0.040±0.010	0.880

[1]TSS, transcription start site.

Table S2. The information of top 100 expression-correlated genes of *CYP24A1* from GEPIA2

Gene Symbol	Gene ID	PCC
FCN3	ENSG00000142748.12	0.62
IGFBP1	ENSG00000146678.9	0.62
CH507-152C13.3	ENSG00000276076.4	0.62
APOC4-APOC2	ENSG00000224916.8	0.62

CRYAA	ENSG00000160202.7	0.62
APOC2	ENSG00000234906.8	0.61
PRAMEF8	ENSG00000182330.10	0.61
PLGLB1	ENSG00000183281.14	0.61
SAA2	ENSG00000134339.8	0.61
RP11-1151B14.2	ENSG00000267675.1	0.61
RP4-564F22.6	ENSG00000275285.1	0.61
RP11-622A1.2	ENSG00000250436.1	0.61
C4BPA	ENSG00000123838.10	0.61
SLC22A1	ENSG00000175003.12	0.61
PLGLA	ENSG00000240935.6	0.61
CTD-3128G10.7	ENSG00000276980.1	0.61
CFHR3	ENSG00000116785.13	0.61
SAA1	ENSG00000173432.10	0.61
GBP7	ENSG00000213512.1	0.61
SAA4	ENSG00000148965.8	0.61
FAM99B	ENSG00000205865.4	0.61
SERPINF2	ENSG00000167711.13	0.61
LRCOL1	ENSG00000204583.9	0.61
RDH16	ENSG00000139547.7	0.61
ASGR2	ENSG00000161944.16	0.6
FMO3	ENSG00000007933.12	0.6
CCND2P1	ENSG00000256847.1	0.6
GYS2	ENSG00000111713.2	0.6
AOX1	ENSG00000138356.13	0.6
TF	ENSG00000091513.14	0.6
PLGLB2	ENSG00000125551.18	0.6
CA5A	ENSG00000174990.4	0.6
F2	ENSG00000180210.14	0.6
SLC13A5	ENSG00000141485.15	0.6
HAMP	ENSG00000105697.7	0.6
ITIH3	ENSG00000162267.12	0.6
DIO1	ENSG00000211452.10	0.6
AMBP	ENSG00000106927.11	0.6
CCL16	ENSG00000275152.4	0.6
LPA	ENSG00000198670.11	0.6
PLG	ENSG00000122194.18	0.6
APOA5	ENSG00000110243.11	0.6
CYP8B1	ENSG00000180432.5	0.6
INHBE	ENSG00000139269.2	0.6
CPN2	ENSG00000178772.6	0.6
APOC4	ENSG00000267467.3	0.6
HAO1	ENSG00000101323.4	0.6
ORM2	ENSG00000228278.3	0.6
ANGPTL3	ENSG00000132855.4	0.6
LECT2	ENSG00000145826.8	0.6
APOA2	ENSG00000158874.11	0.6
CPB2	ENSG00000080618.13	0.6

CFHR4	ENSG00000134365.12	0.6
CYP1A2	ENSG00000140505.6	0.6
INHBC	ENSG00000175189.3	0.6
C19orf80	ENSG00000130173.13	0.6
AHSG	ENSG00000145192.12	0.6
SLC22A10	ENSG00000184999.11	0.6
F13B	ENSG00000143278.3	0.6
HP	ENSG00000257017.8	0.6
CFHR5	ENSG00000134389.9	0.6
AKR1D1	ENSG00000122787.14	0.6
FGB	ENSG00000171564.11	0.6
ITIH2	ENSG00000151655.17	0.6
FGA	ENSG00000171560.14	0.6
C8B	ENSG00000021852.12	0.6
C9	ENSG00000113600.10	0.6
FGG	ENSG00000171557.16	0.6
MBL2	ENSG00000165471.6	0.6
PGLYRP2	ENSG00000161031.12	0.6
F9	ENSG00000101981.10	0.6
C8A	ENSG00000157131.10	0.6
ACSM2B	ENSG00000066813.14	0.6
CYP2A6	ENSG00000255974.6	0.6
CYP4A11	ENSG00000187048.12	0.6
CRP	ENSG00000132693.12	0.6
SPP2	ENSG00000072080.10	0.6
CTC-505O3.2	ENSG00000248709.1	0.6
ALB	ENSG00000163631.16	0.6
HRG	ENSG00000113905.4	0.6
SLC25A47	ENSG00000140107.10	0.6
SLC17A2	ENSG00000112337.10	0.6
CYP4A22	ENSG00000162365.11	0.6
SERPINC1	ENSG00000117601.13	0.6
APCS	ENSG00000132703.3	0.6
HAO2	ENSG00000116882.14	0.6
GC	ENSG00000145321.12	0.6
RP11-115J16.3	ENSG00000253735.1	0.6
RP11-219C24.6	ENSG00000237700.1	0.6
CRPP1	ENSG00000223603.1	0.6
HAO2-IT1	ENSG00000230921.1	0.6
RP11-101E14.3	ENSG00000233415.1	0.6
OR10J6P	ENSG00000158731.2	0.6
RP11-401E5.2	ENSG00000251177.2	0.6
BNIP3P36	ENSG00000271661.1	0.6
VTN	ENSG00000109072.13	0.6
CFHR2	ENSG00000080910.11	0.6
ITIH1	ENSG00000055957.10	0.6
APOH	ENSG00000091583.10	0.6
INS-IGF2	ENSG00000129965.13	0.6

PCC: Pearson Correlation Coefficient

Table S3. The information of 50 CYP24A1-binding proteins from STRING

Protein	ID
AASDH	ENSP00000205214
CBR4	ENSP00000303525
CDC20	ENSP00000361540
CITED1	ENSP00000401764
CYP24A1	ENSP00000216862
CYP27B1	ENSP00000228606
ECSIT	ENSP00000270517
FAU	ENSP00000431822
FDX1	ENSP00000260270
FDX2	ENSP00000377311
FZR1	ENSP00000378529
MAP3K1	ENSP00000382423
MAP3K14	ENSP00000482657
MAP3K19	ENSP00000376647
MAP3K2	ENSP00000387246
MAP3K3	ENSP00000354927
MRPS2	ENSP00000360850
MRTFB	ENSP00000459626
MYOCD	ENSP00000401678
NDUFAB1	ENSP00000458770
PALB2	ENSP00000261584
RPL11	ENSP00000496250
RPL13	ENSP00000307889
RPL15	ENSP00000309334
RPL18	ENSP00000447001
RPL18A	ENSP00000222247
RPL19	ENSP00000225430
RPL21	ENSP00000346027
RPL23	ENSP00000420311
RPL23A	ENSP00000389103
RPL24	ENSP00000377640
RPL29	ENSP00000294189
RPL31	ENSP00000386717
RPL34	ENSP00000378163
RPL35	ENSP00000259469
RPL37A	ENSP00000418082
RPL38	ENSP00000309830
RPL5	ENSP00000359345
RPS15	ENSP00000466010
RPS19	ENSP00000470972
RPS21	ENSP00000345957
RPS25	ENSP00000435096
RPS26	ENSP00000348849
RPS3	ENSP00000278572

RPS3A	ENSP00000346050
RPS6	ENSP00000369757
RPS8	ENSP00000379888
TP53	ENSP00000269305
UBA52	ENSP00000388107
ZNF254	ENSP00000349494
ZNF785	ENSP00000378642

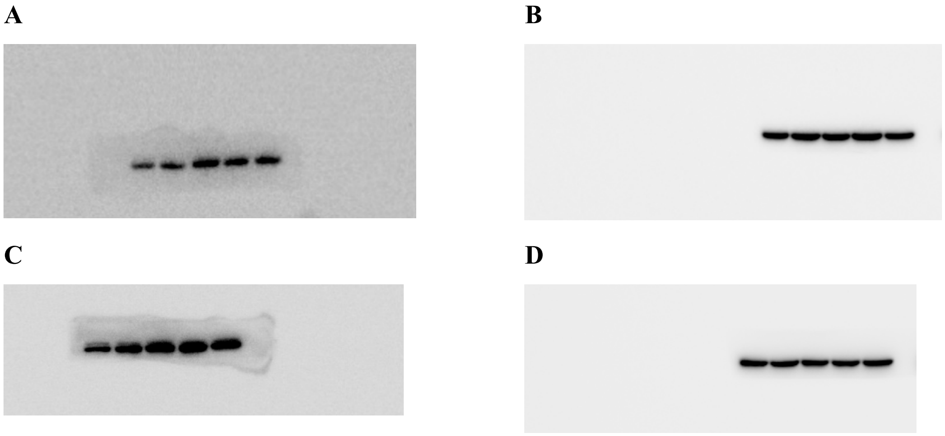

Figure S1. The original western blots of CYP24A1 (**A**) and GAPDH (**B**) for DLD1 cell line, and CYP24A1 (**C**) and GAPDH (**D**) for Lovo cell line.
